# A Deep Feature Learning Method for Drill Bits Monitoring Using the Spectral Analysis of the Acoustic Signals

**DOI:** 10.3390/s18082634

**Published:** 2018-08-11

**Authors:** Caleb Vununu, Kwang-Seok Moon, Suk-Hwan Lee, Ki-Ryong Kwon

**Affiliations:** 1 Department of IT Convergence and Application Engineering, Pukyong National University, Busan 48513, Korea; exen.xmen@gmail.com; 2 Department of Electronics Engineering, Pukyong National University, Busan 48513, Korea; ksmoon@pknu.ac.kr; 3 Department of Information Security, Tongmyong University, Busan 48520, Korea; skylee@tu.ac.kr

**Keywords:** machine fault diagnosis, sound and acoustic processing, pattern recognition, machine learning, deep convolutional autoencoder, deep learning, smart factory, artificial neural network

## Abstract

Machine fault diagnosis (MFD) has gained an important enthusiasm since the unfolding of the pattern recognition techniques in the last three decades. It refers to all of the studies that aim to automatically detect the faults on the machines using various kinds of signals that they can generate. The present work proposes a MFD system for the drilling machines that is based on the sounds they produce. The first key contribution of this paper is to present a system specifically designed for the drills, by attempting not only to detect the faulty drills but also to detect whether the sounds were generated during the active or the idling stage of the whole machinery system, in order to provide a complete remote control. The second key contribution of the work is to represent the power spectrum of the sounds as images and apply some transformations on them in order to reveal, expose, and emphasize the health patterns that are hidden inside them. The created images, the so-called power spectrum density (PSD)-images, are then given to a deep convolutional autoencoder (DCAE) for a high-level feature extraction process. The final step of the scheme consists of adopting the proposed PSD-images + DCAE features as the final representation of the original sounds and utilize them as the inputs of a nonlinear classifier whose outputs will represent the final diagnosis decision. The results of the experiments demonstrate the high discrimination potential afforded by the proposed PSD-images + DCAE features. They were also tested on a noisy dataset and the results show their robustness against noises.

## 1. Introduction

Machine fault diagnosis (MFD) is the process of detecting damages or some abnormal working conditions of the machines using various kinds of signals that they can generate. In the machinery industry, beside many other factors that can affect the productivity, making certain that the machines work properly is surely the primary goal to fulfill. For that purpose, MFD systems are more than necessary in order to avoid breakdowns that can cost in terms of material and human capitals. In the past, MFD studies have been based on methods that consist of composing a model through the analysis of an assortment of parameters from the collected signals [1,2,3]. However, the inherent complexity from the real-world systems and the obvious existence of disturbing factors, such as unwanted noises, make the model based approaches very difficult to handle and not efficient in terms of accuracy. Hence, many researchers have recently adopted machine learning techniques, as we will discuss below.

Machine learning based approaches mainly consist of five steps: data acquisition, feature extraction, which is followed by the evaluation and selection steps of the chosen features, and, finally, the classification process. Collected data from the real-world always contain unwanted elements that can corrupt or disturb the analysis. Furthermore, the data are usually large vectors that contain many redundant observations. The larger is the size of the data to be processed, the more complicated the processing analysis can be. These two difficulties increase the complexity of the classification task for any kind of machine learning algorithms. That is why it is usually admitted to avoid using the raw data as the inputs of the classifier. Instead, we can choose a set of features that strongly characterize the data and use them as the inputs of our chosen classifier. After the feature extraction, evaluation, and selection processes, the final step of the machine learning based MFD consists of the classification, which consists of inferring the operational condition of the machine.

Sound and vibration data have shown effectiveness in the MFD systems of rotating machines, especially because of their capability of carrying the health’s patterns of the tools under investigation. Both kinds of signals, in most studies, are processed with the same methods for detecting the faults. Tandon et al. [4] have reported a detailed review of the different vibration and acoustic methods used for the case of rolling bearing elements.

Statistical features have been widely used in the MFD literature. Kankar et al. [5] have computed six statistical features using the time series of the vibration data of ball bearings, and used them as the inputs of a neural network (NN) based classifier. Saimurugan et al. [6] computed 11 statistical features for the sound and vibration data: they have adopted the six features used in [5] plus five more and given them as inputs to a support vector machine (SVM). Different kinds of statistical features can also be used for the MFD of rotating machines [7,8,9,10,11,12], also computed over the time series of the signals.

Following the demonstration by Lin et al. [13] of the effectiveness of the wavelet decomposition for the fault diagnosis, Kankar et al. [14] have adopted the continuous wavelet transform (CWT) while using the vibrational data of the bearing elements, and the discrete wavelet transform (DWT) was used for the sound based MFD of the car engine by Germen et al. [10]. The wavelet packets (WP) were adopted by Chen et al. [15] and He et al. [16]. Still, as part of the time-frequency domain based features, Wang et al. [17] have used the Hilbert-Huang transform (HHT) as the feature extraction and applied SVM to perform the fault diagnosis of car engine using sound data.

It is quite evident that the time-frequency domain based features are greatly prized in the MFD literature of the rotating machines, especially for their double seizure of the time and frequency domains. The short-time Fourier transform (STFT) also offers such possibility. In the method proposed by Burriel-Valencia et al. [18], STFT was used for the monitoring system of induction machines, like also in the works by Cabal-Yepez et al. [19] and Nandi et al. [20]. Other STFT based MFD systems for the rotating machines can be found in the works by Zhang et al. [21] and Islam et al. [22].

In the MFD literature, what researchers depict as traditional methods usually refers to the model based schemes, like the ones that are referenced in [1,2,3]. Pattern recognition and machine learning based methods are rather referred as data-driven, because of the learning capacities that are incorporated in the scheme. Because we just focus on these kinds of methods, the traditional terminology in this work refers, instead, to the classical schemes that use only the hand-crafted features and do not involve deep feature learning based methods. Since the unfolding of deep learning [23], many works have adopted these automatic high-level feature learning methods, and often combine them with the hand-crafted features. The work by Sohaib et al. [11], for example, presents a hybrid feature learning process, where the time, frequency, and time-frequency domains based statistical features are combined and are given to a stacked autoencoders (SAEs) for a very complex deep feature learning process. Another hybrid feature learning was proposed by Chen et al. [24], with the use of deep belief networks (DBNs). STFT was used as the input of the SAEs by Liu et al. [25]. SAEs were also adopted by Jia et al. [26] while using the raw time series of the vibrational data of rotating machines.

Convolutional neural networks (CNNs) [27] have demonstrated their huge feature extraction capability for the images [28,29]. Since then, they have been widely utilized in the MFD literature. Since the CNNs are designed for images or data represented as images (in a two-dimensional (2-D) representation) and because the time-frequency domain based features can offer this kind of representation, the actual state-of-the-art methods in the MFD have adopted these features and used them with the CNNs for a deep feature learning process. In the work by Verstraete et al. [30], the STFT was used as the input of a deep CNN and Zhao et al. [31] have adopted the WP as the inputs of a deep residual network (DRN). The present paper is mainly focused on the frequency domain based analysis. Unlike vibrations, sound-based analysis provides the advantages of non-contact and non-disintegration, for the signals can be captured independently of the location of the machine under investigation. Vibrations and others acoustic emission based sensors must be in contact with the machines, which can affect the signals being collected [17]. Also, unlike with vibrational data, we demonstrate here that the frequency components of the sounds that are generated by the drills, when processed efficiently, can outperform the time-frequency domain based features.

Drills are tools that are used for boring holes in materials or fastening different materials together. They comprise a perforating or cutting tool attachment, the drill bit, which is gripped by a chuck at one end of the drill and rotates when it is pressed against the targeted material. Despite the fact that rotating machines, like shafts, ball bearing elements, and gearboxes, have been widely studied and discussed in the MFD literature, it is still difficult to find a dissertation that focuses on drills. We propose a diagnosis system that is especially thought and designed for the drilling machines. While the quasi totality of researches only focus on detecting the fault types, the first contribution of the present work is to add one more instance to be detected: the idling time of the drilling machines.

The second and principal contribution is to represent the one-dimensional (1-D) power spectrum of the sounds as images and apply on them some transformations in order to emphasize the distinctive characteristics of each one of the considered condition states. The spectrum imagining, the fact of representing the spectrum data as images, was utilized by Amar et al. [32] and Ciabattoni et al. [33] for the monitoring of the vibrational data of bearing elements and it was used as the principal feature extractor of their methods. Moreover, their 2-D representation consists of stacking the magnitude spectrum of many different data on top of each other in order to construct one single spectral image. Our contributions can be summarized as follows. First, the power spectrum density functions (PSD) of the sounds are extracted. The 1-D vectors containing the logarithmic representation of the PSD of every single data sound are segmented in different windows over the frequency line in such a way that they can fit into a matrix. The created matrices are linearly converted into 8-bit grayscale images and we apply histogram equalization on them in order to uniformly spread all over them the machine’s states related information that is contained inside the power spectrum patterns. The third contribution of our scheme is to consider the obtained images, referred in this work as the “PSD-images”, as the inputs of a deep learning based feature extraction process. A deep convolutional autoencoder (DCAE), which consists of stacked backwards convolutional layers that are used to reconstruct the original PSD-images by up-sampling the network’s latent representations, is adopted in this work for a high-level feature extraction process. Those latent representations that are trapped in the middle of the DCAE are extracted and used as the final feature representation of the original sounds.

**Figure 1:**
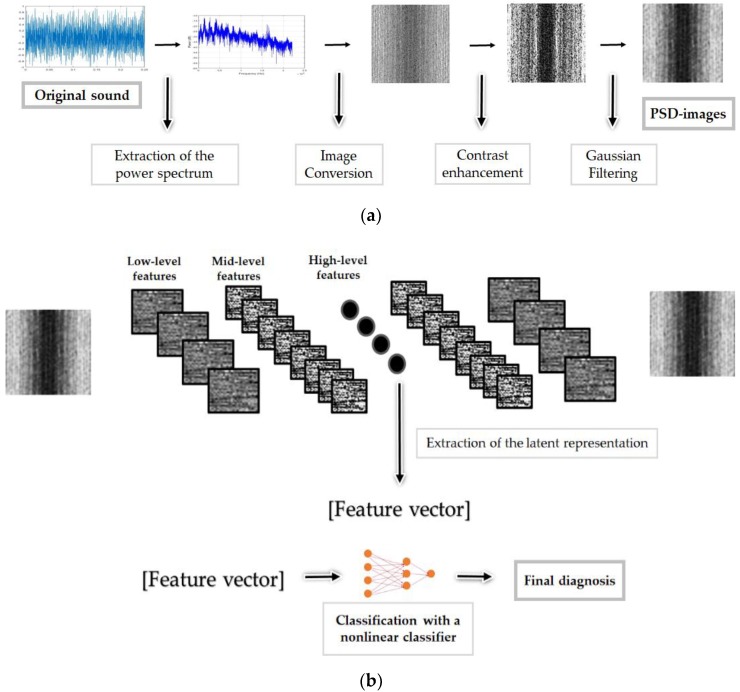
The schematic representation of the proposed fault diagnosis method: in (**a**), we have the building of the power spectral density (PSD)-images, and in (**b**) the deep convolutional autoencoder (DCAE) based feature learning process using the PSD-images, and the final part consisting of the nonlinear classification.

In the last part of the process, the “PSD-images + DCAE” features will be given to a nonlinear classifier whose outputs will represent the final diagnosis. We demonstrate with the results that the image representation and the contrast enhancement of the power spectrum components, the PSD-images, harmonize each class and tend to produce features that are highly discriminative. Figure 1 represents schematically each step of the proposed system. Meanwhile, we conduct a comparative investigation of different feature extraction methods, including traditional and state-of-the-art, which are mostly based on the time or the time-frequency domain, with our proposed PSD-images representation.

The rest of the paper is organized, as follows. Section 2 describes succinctly the problem treated in this paper by defining the different machine conditions that are considered and the motivation beyond this atypical formulation. Section 3 goes through the step by step description of the proposed feature extraction method, while Section 4 addresses the results that were obtained by applying our method, tests other forms of dimensionality reduction, and provides a comparative study with other methods.

## 2. Machine Working Condition Types

Generally, the researches on fault diagnosis systems only focus on detecting the machine’s health conditions, the task being limited to detect healthy and faulty states. In that case, the pattern recognition problem consisting of classifying different objects to different instances is limited to only two classes, or it can be expanded when different faulty conditions are considered. The problem exposed in this work is quite different. We aim not only to detect defected drills but we want also to recognize the sounds produced by the machines during their idle stage. We want to predict whether the sound was generated during the idle or the active time of the machinery system, and also to predict whether the sound that was produced during the active time was generated by the healthy or the faulty drill.

**Figure 2:**
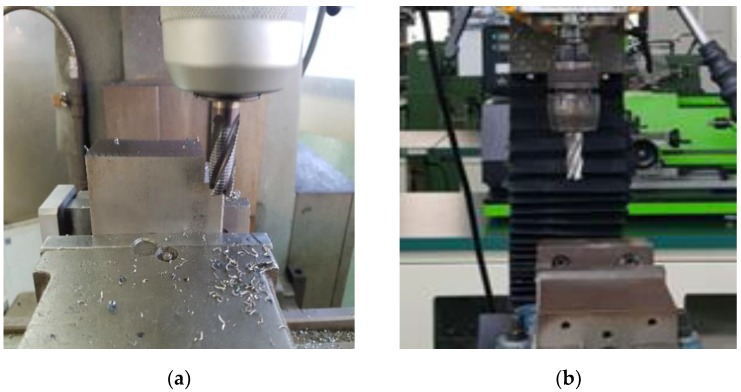
Experimental setup: (**a**) a drill during the active time and (**b**) a drill during the idle time.

We have collected data sounds from healthy and faulty drills during their idling and active time. In this work, the idle time refers to the moment when the drill’s bit, still attached to its chuck, continues to rotate without cutting or boring holes in the materials. The sounds that are produced by both healthy and defected drills during this time are supposed to be similar, or, we should assert, we consider them as from the same state, the idling state, because we want to automatically recognize whether the system is idling, regardless of the health condition of the drill being rotated. Once the system becomes active, which means, when the drills start perforating or cutting the material, they produce sounds that are supposed to be manifestly related to their health condition. It is only at that moment that sounds that are generated by healthy and faulty drills are considered as from different states, the normal and abnormal states. In Figure 2, we show an illustration of the idling and active states from our experimental setups. In Figure 2a, we have a drill that is cutting a steel while rotating, and in Figure 2b, we have a drill rotating without cutting or perforating any material.

The need of detecting the sounds that are produced by the drills during their idling stage is justified by the necessity of having an overall control over the whole machinery system. In the industry sites, for those who have adopted the smart factory concept, most of the machinery systems nowadays work autonomously. The operators can supervise all of the system without the imperative of being right in the places where the machines are located. This fact obviously leads to the exigency of developing a system that can provide a complete remote control. The operators must be able to know whether the system is idling or working even by being far from it. All of the control options can be embedded to the assessment system in order to provide a complete and dynamic scheme: a scheme that is capable of performing diagnosis and also providing information about the working state (active or idling) in the same time.

**Figure 3:**
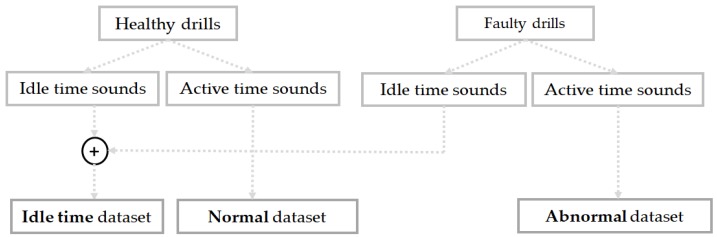
Representation of the datasets considered in this work: the idle time dataset comprises the sounds from both healthy and faulty drills during the idling stage.

**Figure 4:**
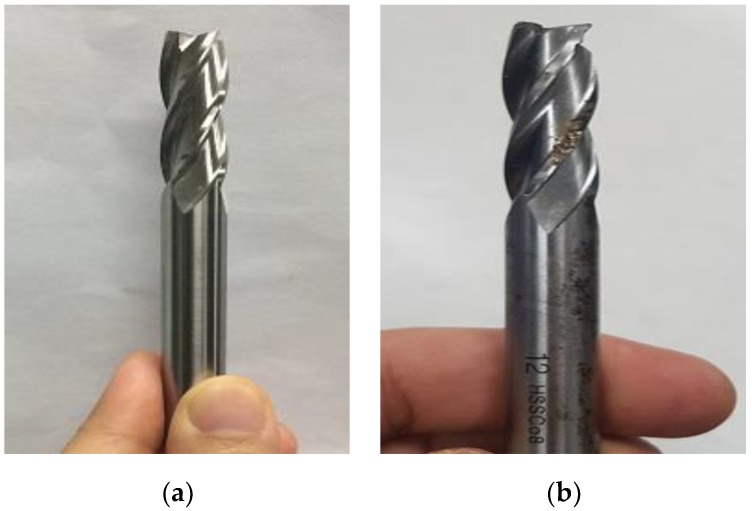
Illustration of drills’ bits: (**a**) healthy drill; and, (**b**) faulty drill with wear.

So, we have collected three different kinds of data in our database according to the three machine’s working condition states described above: the idle sounds, which come from both of the drills during the inactive stage; the normal sound data, collected from only the healthy drills during the active stage; and finally, the abnormal data collected using the faulty drills during the active stage of the machinery system. Only one faulty condition is considered in this work: the wear located on the drill’s bit. Following the fact that only one fault is considered, the pattern recognition problem that we have in this discussion is circumscribed to a classification task with three different instances: idle, normal, and abnormal. Figure 3 illustrates how we have built the different datasets that are considered for our diagnosis system. Next, in Figure 4a,b, we show, respectively, the bits from the healthy drill and the one from the damaged drill that is characterized by an ostensible wear.

**Figure 5:**
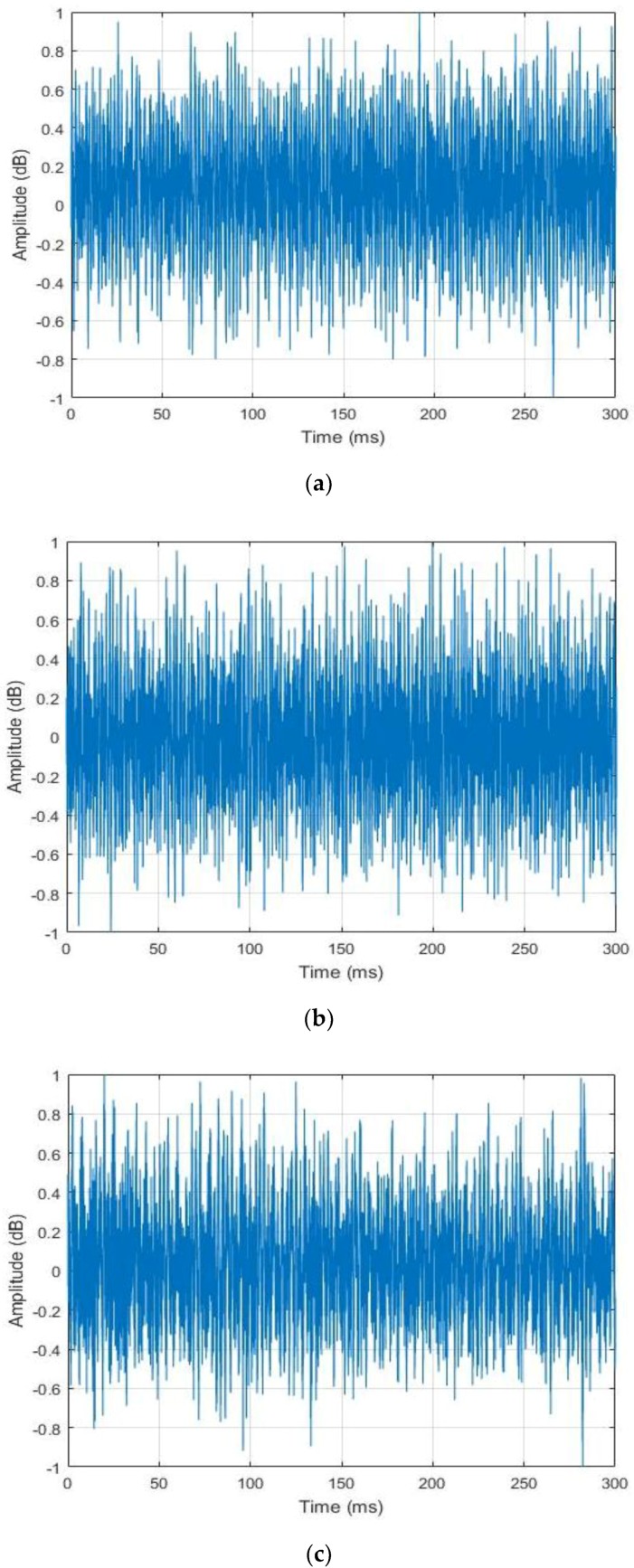
Example of the data samples collected for the experiments: (**a**) idle data; (**b**) normal data; and, (**c**) abnormal data. Each data has a length of 300 milliseconds.

From this point of the paper, all of the sounds from the idle time, normal and abnormal datasets will be, respectively, referred as the idle, normal and abnormal data or sounds. Even from a quick auditory inspection, we can come to the conclusion that the idle data relatively differ from the two active data, normal and abnormal. As the background noises of the experimental rig was not estimated in order to mimic as far as possible the industry site, the collected data show similar random processes in their shapes, as we can notice by analyzing their waveforms that are shown in Figure 5 (for the visualization purpose, the data in the figure were all normalized between −1 and 1). These time series do not expose the inherent differences that exist between the data from the three different states. The purpose of this work is to find a feature extraction method that can reveal the data’s hidden patterns that are related to the machine’s state and health condition. Figure 5 shows the different acoustic signatures of the drills for the three different states.

## 3. Proposed Method

### 3.1. Construction of the Power Spectral Density-Images

Using the time series of a discrete function f(n) containing *N* sampling points, the frequency spectrum F(k), containing also *N* discrete points, is given by the following equation:

(1) $$F\left(k\right)=\sum_{n=0}^{N-1}f{\left(n\right)}^{-j2\pi kn/N}\;\left(k=0,\;1,\;\ldots ,\;N-1\right),$$

where the values *k* represent the frequency components. The PSD can be estimated by computing the power spectrum of every frequency component *k* from Equation (1) using the following equation:

(2) $$P\left(k\right)={F{\left(k\right)}_{real}}^{2}+{F{\left(k\right)}_{imag}}^{2},$$

where $F{\left(k\right)}_{real}$ and $F{\left(k\right)}_{imag}$ represent, respectively, the real and imaginary parts of the frequency spectrum. After this computation, the *k*-dimensional (also *n*-dimensional) vector P(k) contains the power spectrum components of every single frequency value *k*. The idea is to represent this 1-D vector as an image, thus, to “transform” it into a 2-D matrix. The exact emplacement of each frequency value is not important here, since all the data will have, *in fine*, the same shape. We segment the 1-D vector into different windows over the frequency line and position the obtained segments in a column fashion in order to obtain the matrix representation. Let *p* denote the number of segments that we want to obtain. We then divide P(k) into *p* segments of length *l*, with k=l×p, *k* being the original length of P(k), and *l*, the length of the segments. As mentioned before, these obtained segments are positioned as columns and concatenated in order to obtain the l×p matrix representation of P(k). The details about all of the parameters’ value are given in the experimental results section.

**Figure 6:**
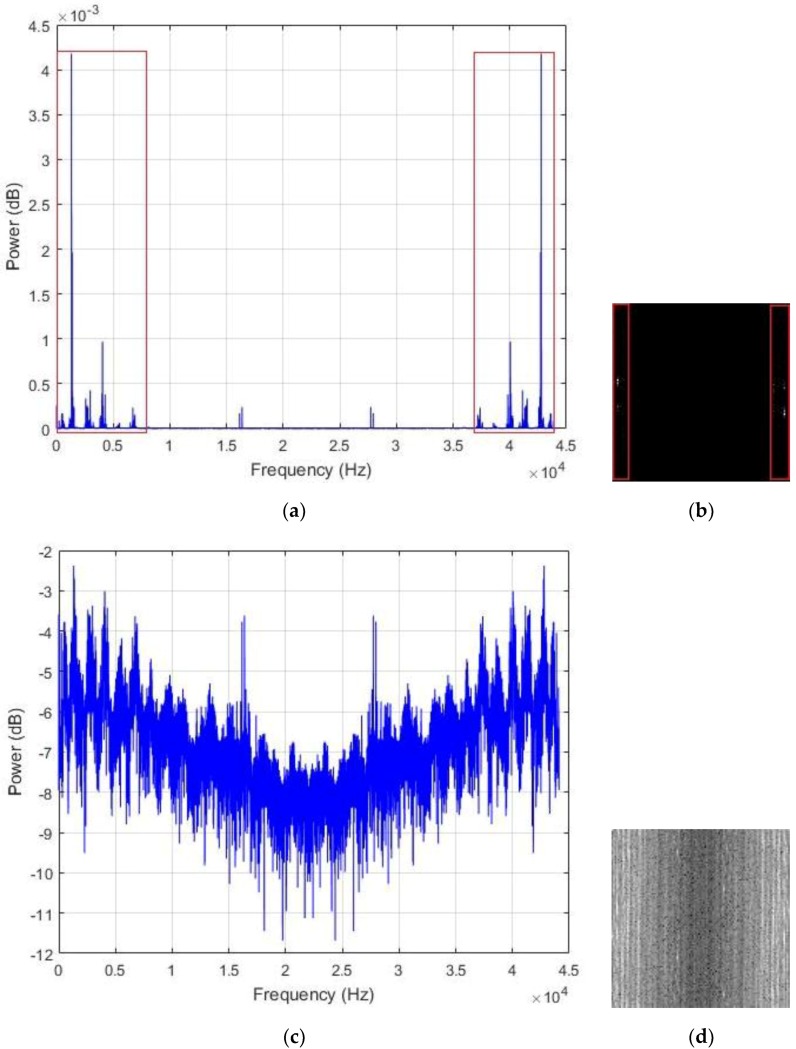
(**a**) The power spectrum of an example data where the low frequency regions with maximal energy are marked in red. In (**b**), the corresponding image, with the low frequency bands also marked in red. In (**c**), we have the logarithmic scale representation of the power spectrum, and in (**d**), its resulting image.

In Figure 6a, we show the typical shape of the PSD encountered with our data sounds. As we can notice, most of the energy is concentrated in the low frequency band. Following the fact that only half of the frequency values are used for the computation of the transform in Equation (1) as a consequence of the Nyquist rate, the power spectrum components are replicated in the two halves of the vector. Using a non-shifted representation, the low frequency bands are represented in the two opposite sides of the frequency line, as we can see with the regions that are marked in red in Figure 6a. When applying our matrix representation to this kind of vector, we also expect the low frequency bands, which are the regions with maximal energy, to be positioned at the two extremities of the matrix.

Figure 6b shows the resulting image that is created by uniformly normalizing the values in the PSD vector by setting the maximum and minimum values to 255 and 0, respectively. Because most of the components in the vector are really small, and due to the significant differences between the peak values and the rest of the vector, most of the spectral elements are represented by the black pixels (the zero or close to zero values) and only the parts corresponding to the peaks and located in the low frequency bands tend to be white, giving the images with very poor contrast, such as the one portrayed in Figure 6b. To avoid such big variance inside the vector and in order to provide a fair contrast to the images, we opt for the logarithmic scale representation of the PSD, as shown in Figure 6c, and the resulting image using this representation is shown in Figure 6d. This final image shows how the energy is distributed in the power spectrum vector, with the center part of the image, which represents the high frequency values, containing most of the dark pixels.

**Figure 7:**
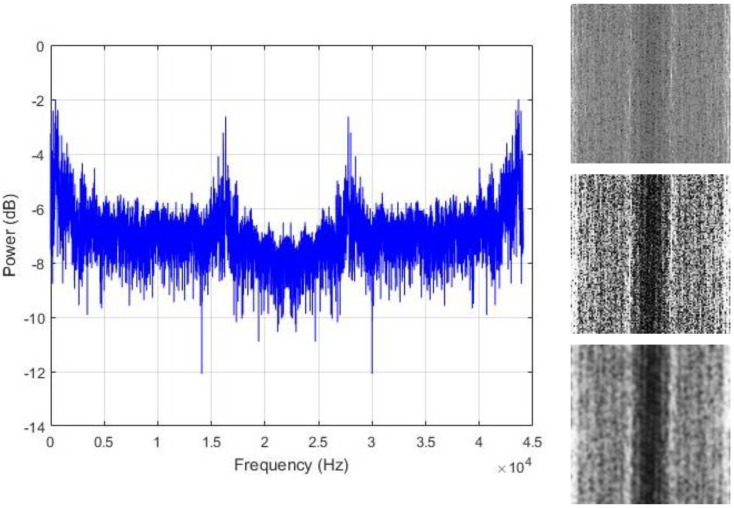
Left column: the power spectrum of an idle data sound; right column: the corresponding PSD-images. From top to bottom: the original image, the histogram equalized and the smoothed versions, respectively. The original size of the PSD-images shown here is 115 × 115.

**Figure 8:**
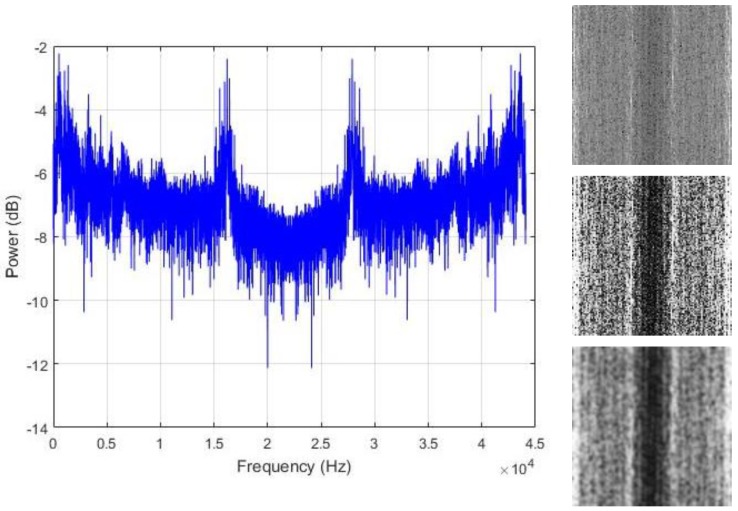
Left column: the power spectrum of a normal data sound; right column: the corresponding PSD-images. From top to bottom: the original image, the histogram equalized and the smoothed versions, respectively. The original size of the PSD-images shown here is 115 × 115.

**Figure 9:**
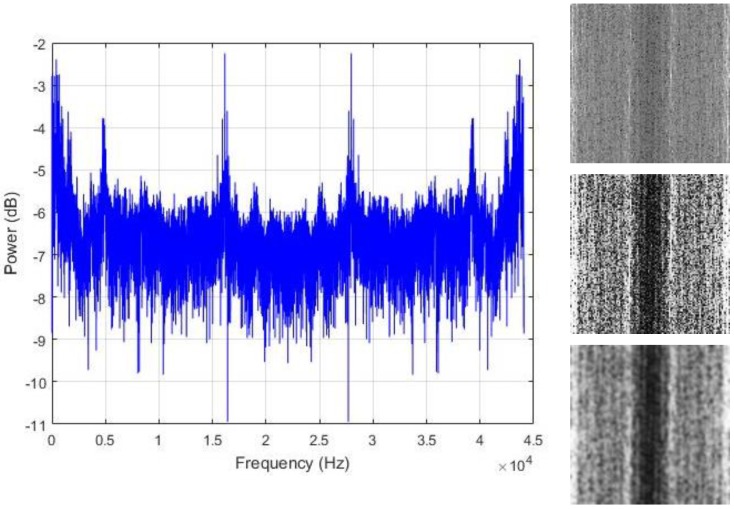
Left column: the power spectrum of an abnormal data sound; right column: the corresponding PSD-images. From top to bottom: the original image, the histogram equalized and the smoothed versions, respectively. The original size of the PSD-images shown here is 115 × 115.

The assumption that is made in this work is that the regions where the energy is maximal are the area that actually contains the useful information that we need in order to discriminate the data. Thus, we propose to spread all over the image those hidden patterns in order to coarsely homogenize the data from the same class. We adjust the contrast of the images with histogram equalization for that purpose. The resulting contrast enhanced images show significantly sharpened edges, thus, increasing the presence of the high frequency components. The final step is to smooth the images in order to reduce the sharpness of the gray variations. The resulting contrast adjusted and smoothed “PSD-images” will represent the inputs that we will use for the final feature learning process. Figure 7, Figure 8 and Figure 9 show some examples of the created PSD-images for the idle, normal, and abnormal data, respectively. The original size of the created PSD-images shown in Figure 6, Figure 7, Figure 8 and Figure 9 is 115 × 115.

### 3.2. Feature Extraction with the Deep Convolutional Autoencoder

Auto-encoders [34,35] are neural network based unsupervised learning methods that are used for the purpose of feature extraction and dimensionality reduction. They comprise nonlinear encoding and decoding processes that can be stacked together. The encoder takes an input x of dimension *d*, and maps it to a hidden representation y of dimension *r*, with usually r<d, using a nonlinear function *f*, such that:

(3) y=f(Wx+b),

where the parameters **W** and **b** are the weights and bias that must be learned by the encoder. The decoder takes the encoder’s output **y** and uses the same function *f* in order to reconstruct the original input **x**. Let **z** denote the output of the decoder, we have:

(4) $$z=f\left(W^{\prime }y+b^{\prime }\right),$$

where the parameters **W′** and **b′** are the weights and bias that must be learned by the decoder. The final solution of the system is given by:

(5) $$\left(W,\;W^{\prime },\;b,\;b^{\prime }\right)=\underset{W,\;W^{\prime },\;b,\;b^{\prime }}{\mathrm{argmin}}L\left(\mathrm{xz}\right),$$

where L(xz) denotes a cost function estimating the difference between the original input **x** and the reconstruction **z**, a difference that we must minimize.

This encoding-decoding process can also be accomplished by using the CNN, giving the so-called DCAE. Unlike conventional neural networks, whose output’s size exclusively depends on the user (the output’s size does not depend on the computations performed by the network, since you can fix the number of the output neurons accordingly to what you need), CNNs are designed to be scale-invariant, which leads to a systematic down-sampling process being incorporated in the architecture and performed by the so-called “pooling layers”. According to the parameters that are adopted during their computations, the convolution mechanisms can also decrease the input’s size. Thus, for the reconstruction process, we need an up-sampling mechanism that will bring us back to the original input’s size.

This can be accomplished by the use of transposed convolution and up-sampling also referred as “deconvolution” and “unpooling” in order to denote the backwards operations. The transposed convolutional layers compute the normal convolution operations over the input, but with the exigency of providing an output volume spatially larger than the input volume [36].

**Figure 10:**
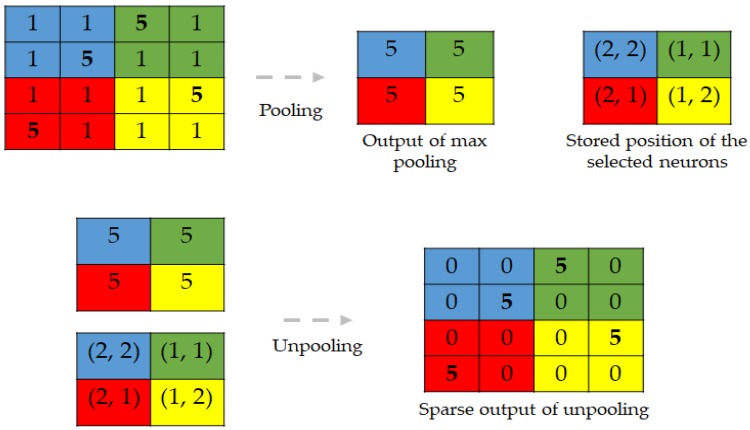
Illustration of pooling with “position storage” and unpooling.

Conventional pooling operators are used to remove some activations that are not important for the discrimination of the data by forcing the network to retain only the neurons that are “strongly” activated inside a given receptive filed. These kinds of operations, even though they help to filter out the noisy activations in the case of very deep networks, they also cause a loss of information in a pixel level that may be critical for a reconstruction. In fact, by increasingly down-sampling the inputs layers after layers, the information related to the exact position of the pixels are progressively lost the more that we go through the network. Thus, to avoid this situation, we store the positions of each selected activation during the maximum pooling operations in the encoding network. During the decoding process, we place each activation to the stored position, and set all of the remaining values to 0. This allows for the unpooling layer to up-sample the input by incorporating the location related information. But, the volume reconstructed by the unpooling layers will be characterized by sparsity, explained by the fact that most of the neurons in the receptive field will be zero. The deconvolutional layers will densify these sparse activations. Figure 10 gives an illustration of the process of pooling and unpooling for the DCAE.

As illustrated in the figure, the positions that are stored during the max pooling operation will be used during the unpooling process in order to enable a pixel level accurate reconstruction. This form of unpooling process is also made feasible by the fact that every single encoding layer has its corresponding decoding layer, in order to respect the symmetry exigency of the decoding-encoding process.

**Figure 11:**
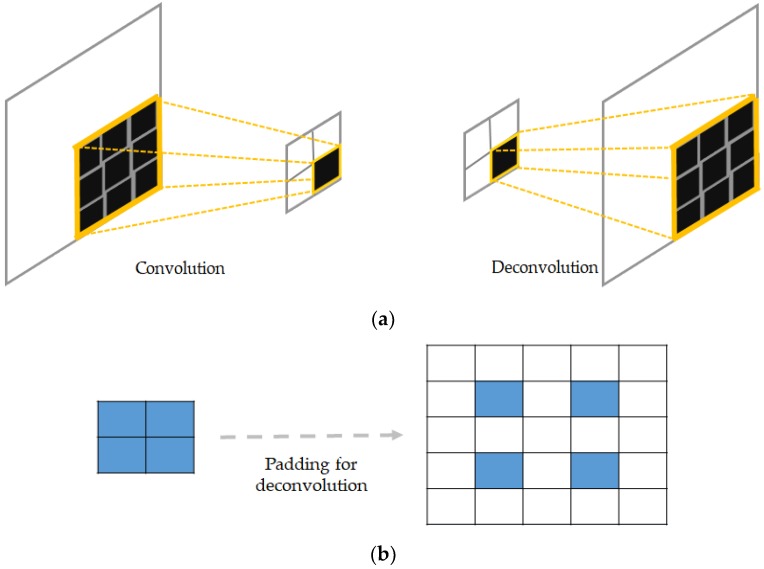
(**a**) Illustration of convolution and deconvolution; and, (**b**) illustration of padding for backwards convolution (deconvolution).

As we can notice in Figure 10, the output from the unpooling layer still remains a sparse representation of the previous layer that underwent the max pooling. In the DCAE, the decoder starts with an unpooling layer, and every single unpooling layer is systematically followed by the deconvolutional layer whose task will especially be to densify the sparse representation that is inherited from the unpooling operator. Unlike conventional convolutional operators, which take multiple entries and output a single value, the deconvolutional layers connect a single activation with multiple outputs. This can be done by padding some “invisible” window around the concerned neuron in order to perform the convolution-like operations. Figure 11 illustrates how the deconvolution works. In Figure 11a, we show the difference between a conventional convolutional layer that is used in the encoding process and the corresponding deconvolutional layer that is used in the decoder. We can remark that, while the first operation reduces the size of the input, the latter somehow reconstructs the original size. In Figure 11b, we show an example illustrating how padding can be accomplished in case of deconvolution. The original input is shown in blue, and the padding neurons are used in such a way that the final output is enlarged after the computations. Note that these computations will finally replace the zero values that were previously obtained with the unpooling operation; thus, densifying the sparse representation from the unpooling.

Every convolutional layer will have its corresponding deconvolutional layer. The outputs from two corresponding layers must agree in size and volume (the number of feature maps), and thus, cropping can be done in order to force all of the deconvolutional layers in the decoder to output a volume of the same size with their corresponding convolutional layers in the encoder. The network first starts by down-sampling the input while extracting high-level features and when we reach the layer where the latent representations will be located, the backwards operations start until the original size is reconstructed. The adopted cost function in this work is the cross-entropy cost [34], described as:

(6) $$L\left(\mathrm{xz}\right)=\sum_{i=1}^{N}[x_{i}\log z_{i}+\left(1-x_{i}\right)\log\left(1-z_{i}\right)],$$

where *N* represents the total number of data, **x** is the original input, the PSD-image, and **z** the output of the decoder. The network learns the parameters in Equation (5), so that the error in Equation (6) is minimized. Finally, the latent representations trapped in the middle of the network will be the final features. They will be given to a nonlinear classifier for the final discrimination step. We also demonstrate in this same time that the proposed features are highly discriminative in the sense that any nonlinear classifier can find a way to separate them.

While the final classification step is one of the most important parts, if not the most important, in the MFD systems, the purpose of this work is first to show how the features created by the DCAE, and, most importantly, learned from the PSD-images provide a better discrimination potential than most of the referenced feature representation methods for the separation of the three machine states that are defined in this work. The next section discusses, in detail, the obtained results of the proposed method.

## 4. Results and Discussions

### 4.1. Experimental setup

In Figure 2, we show the experimental setup for the collection of the data, and in Figure 4, we show the drilling tools used for the experiments. We have collected the data sounds while using a microphone. Each type of drills was used for the collection of the idle data during the inactive stage, healthy drills were used for the collection of the normal data and the faulty drills were used for the acquisition of the abnormal data, as clearly explained in Section 2 (see Figure 3). During the measurements, the following data acquisition parameters were used: the signal length was set to be 300 milliseconds, as we can see for the data samples shown in Figure 5, and the sampling frequency used was 44.1 kHz. With these parameters, every single data in our database represents a vector containing 13,230 elements. The number of data samples for each dataset is 798, 1144, and 1051 for the idle, normal, and abnormal datasets, respectively, which gives a total of 2993 data sounds.

The tools used are cutting drills HSSCo8, which stands for high speed steal with 8% cobalt. The diameter of the drill’s bit is 12 mm. The rotation speed of the drill’s bit was set to 1000 rpm for the collection of the three states and the workpiece shown in Figure 2a and used during the cutting time is also high speed steal with 8% cobalt.

### 4.2. Results

We start our scheme by extracting the power spectrum of the data, as explained in Section 3. Some of the PSD from our samples are shown in Figure 7, Figure 8 and Figure 9. We have used all of the PSD components in a non-shifted fashion. As the data are 13,230 dimensional vectors, and by using Equation (2), every single PSD vector contains also 13,230 elements, which provides fairly big vectors to handle. The following step is to build the PSD-images. We have created images of size 115 × 115, as the ones that are used for the illustration in Figure 6 and Figure 7. Giving the fact that each vector data that we have contains 13,230 elements and 115 × 115 (13,225) among them were utilized for the creation of the PSD-images, it must be noticed that only five values in the original PSD vectors were left out in order to construct the square images.

**Figure 12:**
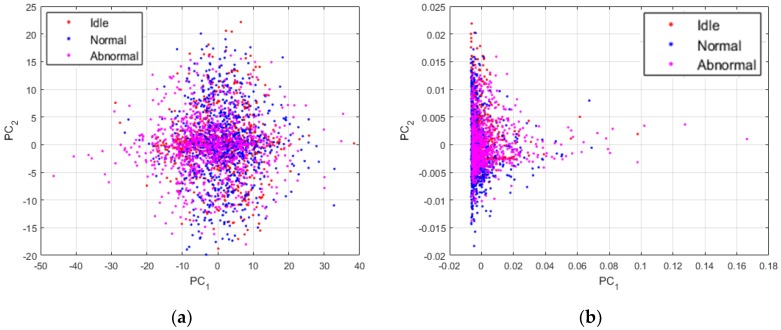
(**a**) Projections of the data using their raw time series; and, (**b**) projections of the data when represented by their power spectrum. There are 2993 data in each figure.

The time series of the data do not show any important information or relevant cues concerning the discrimination of the three machine condition states treated in this work. In Figure 12, we show the plots of the data while using different sorts of representation. All of the projections concerning the visualization of the data were obtained using the principal component analysis [37]. PC1 and PC2 in the images denote the first and second principal components axis, respectively. The idle, normal and abnormal data are shown in red, blue, and magenta, respectively. Also, the data shown in Figure 12 are the projections of all the 2993 data that have been used for the experiments.

In Figure 12a, we show the plots of the original data sounds using their raw time series. As we can remark, the data from the three states are all clustered together, like they were from the same dataset. These projections give us an indication concerning the fact that most of the time domain based analysis will face some significant difficulties in order to find a way for an accurate discrimination of these data. In Figure 12b, we show the plots of the data represented by their power spectrum. Even though our work is mainly based on the assumption that the power spectrum of the sounds manifestly contain the useful information that we need for our diagnosis scheme, we can still notice that the projections of the PSD do not reveal these information. Yet again, the data are clustered together and using them in these conditions will eventually pose certain problems in terms of discrimination accuracy.

**Figure 13:**
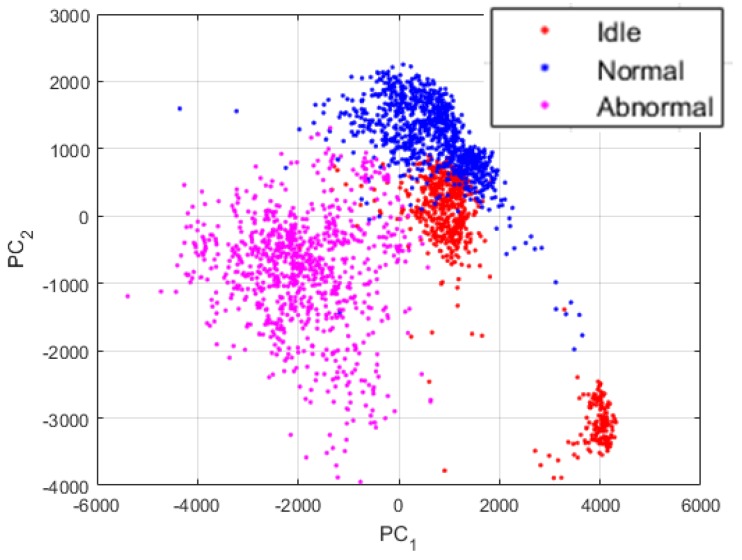
Projections of the data represented by their PSD-images. Also, 2993 data are shown here.

Though, the projections exhibited in Figure 13 were obtained by using the proposed PSD-images feature representation. By analyzing these projections, we can first admit that the differences between the three states were significantly emphasized and exposed by the PSD-images, as we can clearly distinguish three main clusters in the data. The idle data, since they come from drills with different health conditions, also reveal their inherent differences that are denoted in the figure by the two separated clusters of the red dots. Some idle data have unique acoustic signatures that can be explained by the fact that the noises generated by certain drills that show severe damages can be very unstable over time, since the severe wear can cause non-negligible frictions with the chuck while the bit rotates. Yet, the PSD-images manage to relatively homogenize the idle sounds by forcing them to remain in the same projection sub-space. In fact, a good nonlinear classifier can still regroup the projections in Figure 13 in three classes.

The projections that are shown in Figure 13 are really discriminative, as we will explain in the next section where we compare them with other feature extraction methods. Using these projections as the inputs of a neural network based classifier, for example, yields a classification accuracy outperforming most of the time and time-frequency domain based features. But, as we can see in the figure, a big part of the data are still mixed together. In order to provide highly discriminative features, we propose to utilize these PSD-images as the inputs of the DCAE and then adopt the high-level features that will be extirpated from the DCAE as the final data representation, the so called “PSD-images + DCAE” features.

The architecture of the DCAE is shown in Table 1. We recall that the size of the PSD-images are 115 × 115, and the hyper-parameters concerning the two networks were chosen by cross-validating many different hypotheses. We apply three convolution operations, each one of them being followed by a max pooling for down-sampling the input. The first convolutional layer uses 32 filters of size 11 × 11, with a stride window of two and a padding of one. This leads to an output volume with a size of 54 × 54 × 32. We may argue that the input size is considerably reduced, and, thus, causing a significant loss of spatial information that might, thereafter, pose certain problems for the reconstruction process. While this can be true, we must remind that the most important factor to consider, while using these computations is not to finally obtain a fairly precise reconstruction, as it can be required in the case of semantic segmentation problems [36,38,39], where the input data must match with a predefined mask. The most notable gain in the use of the DCAE for our work, since we are not seeking the outputs recovered by the decoder, but for what is happening in the middle of the network, is the recuperation of the nonlinearities provided by the convolution operations of the DCAE with the high-level features trapped inside it.

Moreover, the “storage” system that was used during the pooling operations, as explained in the previous section, will finally allow a reconstruction not very far from the original input. The remaining details of the network’s architecture can be seen in Table 1. As explained in the previous section, the symmetry that is imposed by the encoding-decoding process requires a symmetric network, with each layer in the encoder having its corresponding layer in the decoder. The final convolutional layer, denoted in the table by “Conv3”, is fully connected because its filters’ size is equal to the size of its input volume. “Conv3” layer takes the 6 × 6 × 64 volume outputted by the “Max pool2” layer and applies on it 124 different filters of size 6 × 6. We know that a fully connected layer can be thought of as a convolutional layer with a kernel size that is equal to the input. The output of this “Conv3” layer is a vector containing 124 elements. These elements represent the latent representations that we are seeking for. After this layer, the decoding process is launched by reversing each operation taken in the encoder.

**Figure 14:**
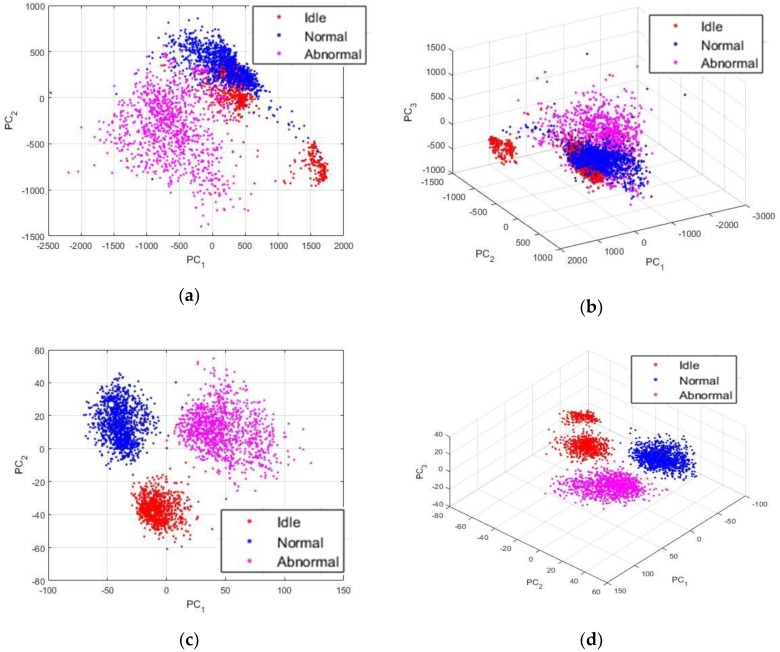
Visualization of the low-level features learned by the DCAE in a two-dimensional (2-D) (**a**) and three-dimensional (3-D) (**b**) view. Visualization of the high-level features in a 2-D (**c**) and 3-D (**d**) view. There 2993 data in the figures.

We show the features that are learned by the DCAE in Figure 14. From this point of the discussion, we will be referring to these learned features as the “PSD-images + DCAE” features. In Figure 14a, we have shown the projections of the activations outputted by the first convolutional layer, the so-called low-level features, using the first two principal components. There are not major changes when we compare them with the projections that are shown in Figure 13, which are the actual inputs of the DCAE. In Figure 14b, we have the same projections using the first three principal components. The 3-D view gives us a better visualization of how plenty of the idle and normal data are mixed together. The mid-level features, the activations from the second convolutional layer, are not shown here since their differences with the projections exhibited in Figure 14a,b are not notable. In Figure 14c,d, we show the high-level features, the activations outputted by the third convolutional layer, i.e., the PSD-images + DCAE features. We can remark how the network has learned highly discriminative features. In Figure 14c, we distinguish clearly three different clusters, as the two groups of the idle data are forced to merge. But, when utilizing the third principal component for the 3-D view shown in Figure 14d, we can clearly notice that we still have two clusters of the idle data, but they are all forced to lay on top of each other, thus, occupying a quite distinctive projection sub-space.

By giving these PSD-images + DCAE features to a classifier, we can expect quite outstanding discrimination results. We have given these elements to a neural network based classifier for the final part of the proposed diagnosis scheme. We recall that the features extracted from the DCAE are 120 dimensional vectors. Thus, the first layer of the network has 120 neurons. Only one hidden layer was adopted, and the final layer comprises three neurons according to the three kinds of data that we want to discriminate, which gives a simple 120-10-3 network’s architecture. The hidden layer uses the hyperbolic tangent as the activation function, and the output layer uses the softmax function whose outputs will represent the probabilities that a given data belongs either to one of the three machine states.

**Figure 15:**
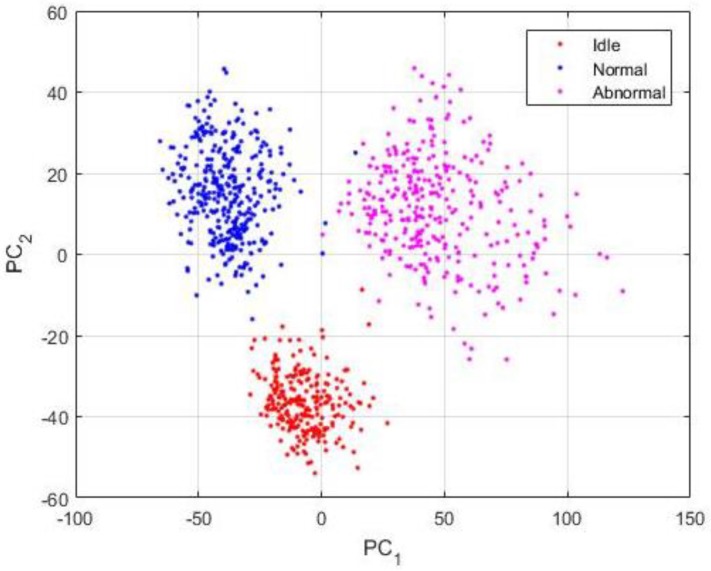
Visualization of the PSD-images + DCAE features of the 897 testing data.

Table 2 shows all of the details concerning the network’s parameters and also the learning process. 70% of the data were used for training the network, the remaining 30% were used for testing the trained network. For the training process: 559 idle data, 801 normal data, and 736 abnormal data were used. For testing the network, we have: 239 idle data, 343 normal, and 315 abnormal data. While in Figure 14 we have plotted the totality of the 2993 samples in the datasets, Figure 15 shows the visualization of the PSD-images + DCAE features of only the 897 testing data.

**Figure 16:**
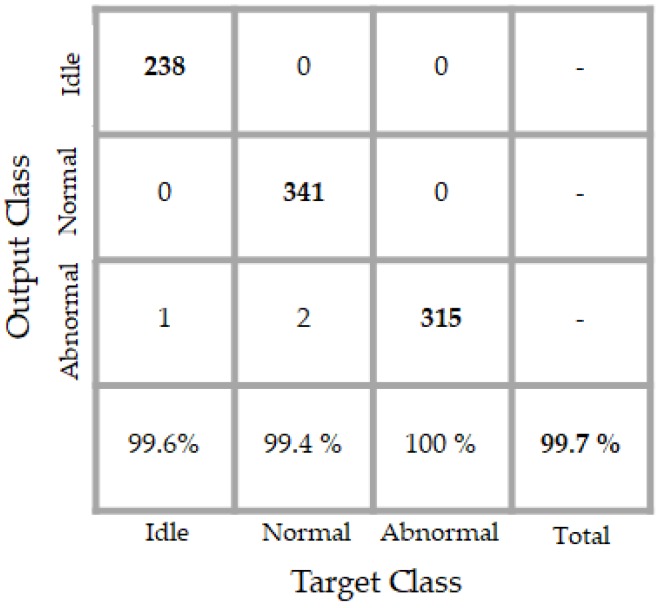
Confusion matrix of the classification results. “Target class” denotes the labels and “output class” denotes the actual results of the network.

The results of the classifier are shown in details in the confusion matrix that is exhibited in Figure 16. The classifier performs an accuracy of 99.7% over the testing data whose projections are depicted in Figure 15. By analyzing the projections in that figure, we can notice that some blue dots (in number of two) appear in the “magenta” (abnormal data) sub-space. When analyzing now the confusion matrix, we see that only two normal data were misclassified as abnormal by the classifier. We see another red point (only one) a bit too close to the abnormal data in the projections. In the confusion matrix, the network has misclassified one idle data as an abnormal one. Meanwhile, the classifier accomplishes a perfect discrimination of the abnormal data, since all of them were recognized as abnormal.

**Figure 17:**
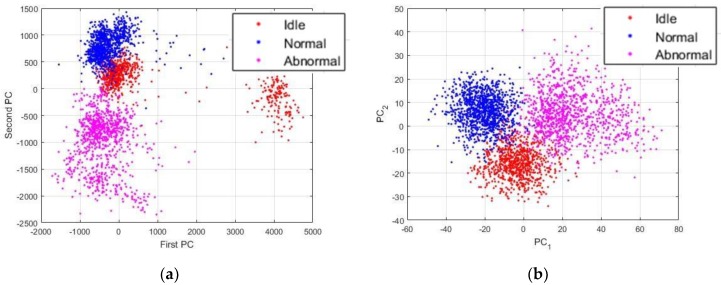
(**a**) Visualization of the noisy dataset when the data are represented by their corresponding PSD-images; and, (**b**) Visualization of the PSD-images + DCAE of the noisy dataset.

In order to test the robustness of the developed PSD-images + DCAE features, we have artificially embedded Gaussian noise in the original dataset. To avoid uniformity in the embedding process, noises were generated randomly in a normal distribution with the signal-to-noise ratio varying between 5 and 10 dB. The goal is to add more complexity in the original data and to see how the proposed features still manage to discriminate the noisy version of the data. In Figure 17, we show the projections of the noisy dataset. In Figure 17a, we show the noisy data that are represented by their corresponding PSD-images, and in Figure 17b, we show the features learned by the DCAE from the PSD-images, the PSD-images + DCAE features. As we can notice, the data are still well clustered.

**Figure 18:**
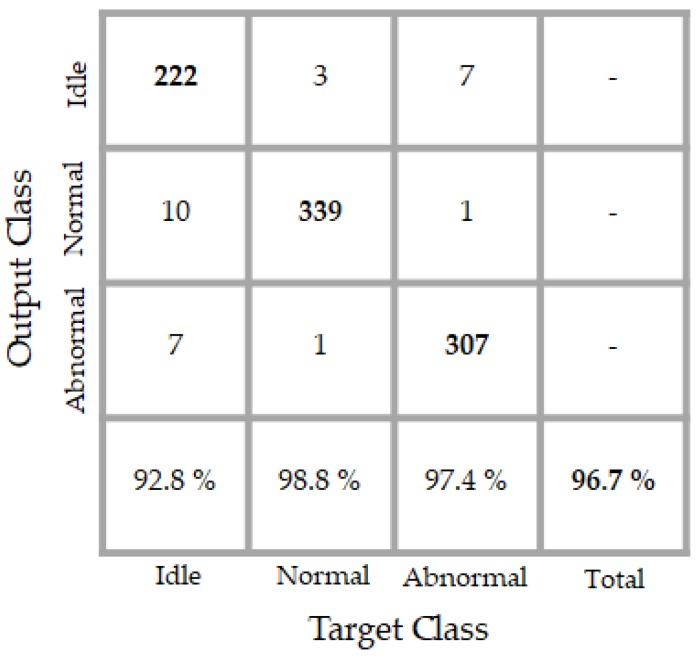
Confusion matrix of the classification results over the noisy test data. “Target class” denotes the labels and “output class” denotes the actual results of the network.

The PSD-images + DCAE features that are shown in Figure 17 were given to our trained network and the classification results are portrayed in the confusion matrix in Figure 18. 17 idle data were misclassified, 10 as normal, and seven others as abnormal. We already discussed the inconstancy of the idle dataset, and the artificially added noises really complicate the discrimination of these data. It is necessary to mention here that the network did not see any of the data from the noisy dataset during the training, but, still manages to discriminate them, thanks to the discrimination potentiality afforded by the PSD-images + DCAE features. In fact, 92.8% of the idle data (222 over 239) were well recognized, which denotes an impressive robustness to noises of the classifier.

The network accomplishes a total of 96.7% of accuracy. We notice that the total accuracy decreases only by 3% while using the noisy dataset. We remind that the quantity of the noises that is added to the original data, i.e., using a signal-to-noise ratio between 5 and 10 dB, is significant, but still, the proposed PSD-images + DCAE features manage to homogenize the data from the same class and strengthens (boosts) the heterogeneity between different classes in the same time.

### 4.3. Comparative Study

The aim of this part is not to provide a quite precise and flawless comparative analysis, since the different methods that will be evoked here were not all designed for the same kinds of signals, as some approaches adopt the vibrational signals and others the ultrasonic data. Moreover, as far as deep learning based methods are concerned, the system’s accuracy can essentially depend on many different hyper-parameters, as ones can be changed from a work to another. The modest goal for this comparative study is to provide an indication of the discrimination potentiality of the different feature learning methods that are widely used in the machine fault detection when they are applied to the sounds generated by the drills and for the critical discrimination of the three machine states considered in this work.

**Figure 19:**
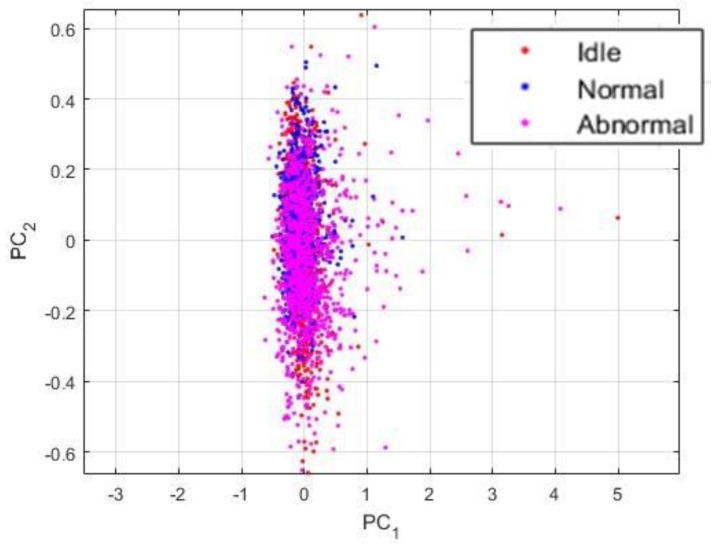
Projections of the data represented by their statistical features.

We start with the traditional statistical features computed using the raw time series of the data as proposed in [7,8,9,10,11,12]. The idea beyond these methods is to find some distinctive descriptors and use them as the inputs of the learning algorithm instead of using the original raw data. We compute the eleven statistical features used in [11,12] over the collected sounds and show their projections in Figure 19.

In Figure 12a, we have shown the projections of the raw time series of the sounds. As in that case, the statistical features computed over the time series do not help to find the differences between the data, as we can remark how all the three classes have even been homogenized in the same fashion in Figure 19. This gives us a hint as to about how the sound data from the three states are statistically correlated. Thus, prior to any analysis, a statistical homogenization of each class and a maximization of their differences in the same time are critically necessary.

**Figure 20:**
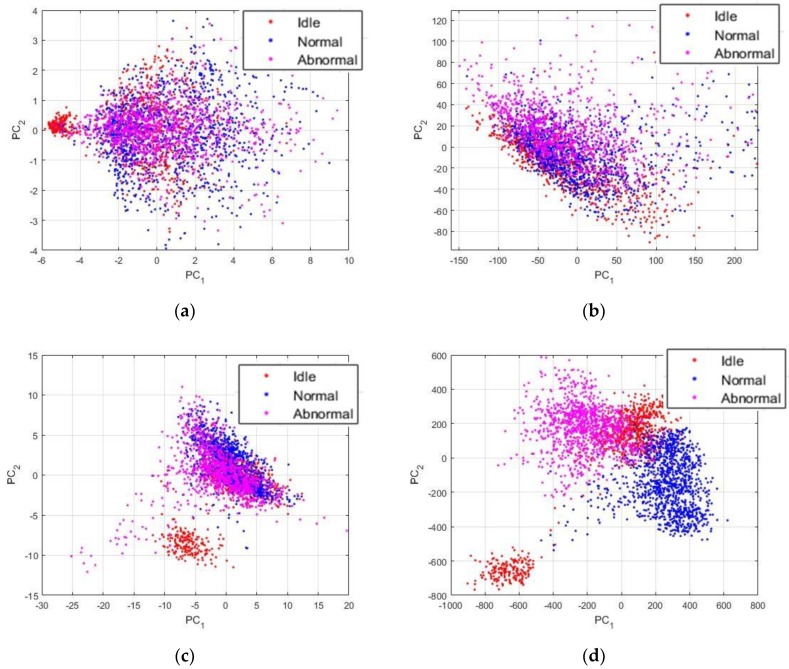
Projections of the data using different feature representation: (**a**) the statistical features computed using the continuous wavelet transform (CWT); (**b**) the Hilbert-Huang transform (HHT) components; (**c**) the wavelet packets (WP); and, (**d**) the short-time Fourier transform (STFT) components.

Though, as part of the time-frequency domain based analysis, we have, quasi exhaustively, the DWT and CWT components, as proposed, respectively, in [10,14], the wavelet packets as used in [31], the HHT components [17], and the short-time Fourier transform, as developed in [30]. It is necessary to mention that in some of these works, the statistical features are computed using these time-frequency domain representations. In Figure 20, we show the projections of these features computed over our data.

In Figure 20a, we have the statistical features that are computed using the continuous wavelet coefficients, as proposed in [14]. Besides the fact that some idle data, surely the ones from the critically damaged machines, are clustered in a distinctive group, the remaining sounds are all mixed. In Figure 20b, the HHT components, as proposed in [17], were used, and, as we can see, there are no meaningful information that were seized from the data. The wavelets packets, as computed in [31], were extracted with the same parameters from our data and are shown in Figure 20c. As we can remark, they also fail to expose the differences between the data, except for the unique idle sounds.

It is not really surprising that the most relevant representation comes from the short-time Fourier transform, as proposed in [30] and illustrated in Figure 20d. In fact, the STFT acts slightly like the power spectrum, but by taking also into account the time parameter. Using the projections shown in Figure 20d, can lead to a quite good discrimination result. If we compare these data with the PSD-images that are shown in Figure 13, we can still remark that the PSD-images have better discrimination potentiality, since in Figure 20d, a big part of the idle data remains clustered with the other two classes.

**Figure 21:**
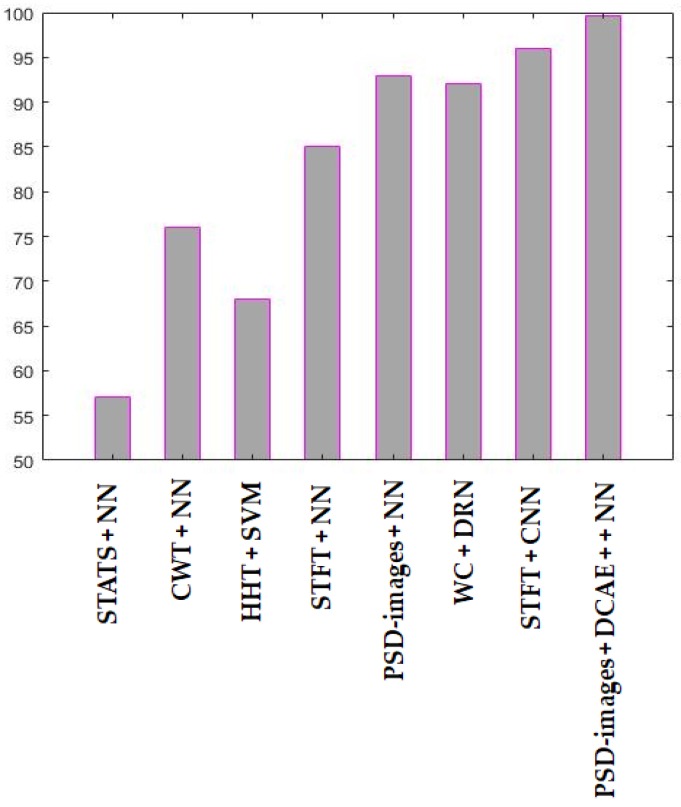
Results of the different approaches used during the experiments. NN: neural network; SVM: support vector machine.

The CWT coefficients were given to a neural network based classifier, the HHT coefficients to an SVM. In [30], they have used the STFT as the input of CNN. Finally, in [31], the wavelet packets were positioned in matrices and were fed to DRN. We show the classification results for all these methods in Figure 21.

As we could expect, the time domain based statistical features, whose projections are shown in Figure 19, perform very poorly. The network could not reach 60% of accuracy. For the CWT and HHT, the classification reaches 76% for the first, with a neural network, and stagnates at 68% for the later, with an SVM. The state-of-the-art deep learning based methods reach outstanding results. The wavelet packets with the DRN stagnate at 92%. The STFT, used as the inputs of a CNN, reaches 96% of discrimination of our data, as we could expect by visualizing the fairly discriminative projections that are shown in Figure 20d.

For a head-to-head comparison between the PSD-images and the STFT, we have given both of them to a shallow network in order to investigate how discriminative they can be when used alone, i.e., without any further deep learning based feature extraction. The PSD-images outperform the STFT by reaching 93% of accuracy. The STFT stagnate at 85%, as many of the idle data were misclassified, as we can clearly notice in Figure 20d. As a conclusion, the PSD-images, even when being used alone, outperform most of the time-frequency domain features.

In Figure 21, the “STATS + NN” stands for the time domain based statistical features used with neural network. “CWT + NN” denotes the continuous wavelet coefficients used with neural network, “HHT + SVM” is the Hilbert-Huang Transform that is used with support vector machines. “STFT + NN” denotes the short-time Fourier transform used with neural network, “PSD-images + NN”, the PSD-images used directly as the inputs of a neural network. “WP + DRN” is the wavelet packets used with a DRN, “STFT + CNN” is the short-time Fourier transform used with CNN. “PSD-images + DCAE + NN” is the proposed diagnosis scheme.

## 5. Conclusions

We have investigated an automatic diagnosis system for the drilling machines while using the sounds that they produce. Unlike the majority of the studies that only focus on detecting the damages, we have presented an original system specifically conceived and designed for the drilling machines, a system able to detect not only the sounds produced by the defected drills, but that is also able to determine whether the sounds were produced during the active or the inactive time of the drilling system. We have proposed a novel feature representation method that extracts the power spectrum components of the sounds, converts them into images, applies contrast enhancement on them, reduces their gray variations with a spatial filtering mask, and uses them as the inputs of a deep convolutional autoencoder for a high-level feature learning process. The obtained results demonstrate how highly discriminative the proposed PSD-images + DCAE features are, and also demonstrate their robustness against noises. In fact, we have massively added noises to our data and demonstrated that the proposed PSD-images + DCAE features still manage to discover and expose the data’s hidden patterns that are useful for their discrimination.

We have conducted a quite representative comparative study by showing the results of the traditional and state-of-the-art methods when applied on our data. We have demonstrated how the PSD-images + DCAE features lead to better discrimination results when compared to the other feature extraction methods. We have also demonstrated, in the same time, how the proposed PSD-images, when used alone without any deep feature learning process, outperform most of the time and time-frequency domain based features.

## Figures and Tables

**Table 1:** Architecture of the DCAE.

Layer ^1^	Filter Size	#Filters	Stride	Padding	Output
Input	-	-	-	-	115 × 115
Conv1	11 × 11	32	2	1	54 × 54 × 32
Max pool1	2 × 2	-	2	0	27 × 27 × 32
Conv2	7 × 7	64	2	1	12 × 12 × 64
Max pool2	2 × 2	-	2	0	6 × 6 × 64
Conv3	6 × 6	124	0	0	1 × 1 × 124
Deconv3	6 × 6	64	0	0	6 × 6 × 64
Unpool2	2 × 2	-	2	0	12 × 12 × 64
Deconv2	7 × 7	32	2	1	27 × 27 × 32
Unpool1	2 × 2	-	2	0	54 × 54 × 32
Deconv1	11 × 11	1	2	1	115 × 115

^1^ “ConvN” denotes the *N*th convolutional layer. “Max poolN” denotes the *N*th maximum pooling layer. “DeconvN” denotes the *N*th deconvolutional layer, and “UnpoolN” refers to the *N*th unpooling layer.

**Table 2:** Details of the classification network.

Number of input neurons	120
Number of output neurons	3
Number of hidden layers	1
Number of neurons in the hidden layer	10
Activation function for the hidden layer	Hyperbolic tangent
Activation function for the output layer	Softmax function
Learning algorithm	Levenberg-Marquardt backpropagation ^1^
Error function	Mean square
Number of training epochs	51
Total number of data	2993
Data used for training	2096 (~70%)
Data used for testing	897 (~30%)

^1^ See [40].
